# Enhancement of Immersive Technology Use in Pediatric Health Care With Accessible, Context-Specific Training: Descriptive Feasibility Study

**DOI:** 10.2196/56447

**Published:** 2024-07-30

**Authors:** Brian S K Li, Brendan Fereday, Ellen Wang, Samuel Rodriguez, Karin Forssell, André N Bollaert, Maria Menendez, Thomas J Caruso

**Affiliations:** 1 Department of Comparative Literature Princeton University Princeton, NJ United States; 2 Department of Anesthesiology, Perioperative and Pain Medicine Stanford University School of Medicine Palo Alto, CA United States; 3 Stanford University Graduate School of Education Palo Alto, CA United States; 4 Invincikids, Inc San Bruno, CA United States; 5 Stanford Children's Health Palo Alto, CA United States

**Keywords:** immersive technology, implementation, adult learning, education, pediatric, accessibility, training, therapeutic, pediatric care, utilization, virtual reality, VR, monitoring, license, development, software, monitoring software

## Abstract

**Background:**

Immersive technology provides adjuncts for pediatric care. However, accessibility and inadequate training limit implementation of this technology. Standardized instruction with no-cost software licensing may improve health care professionals’ facility with immersive technologies.

**Objective:**

This descriptive feasibility study aimed to examine the applications of immersive technologies in pediatric health care, including virtual reality (VR) and projectors.

**Methods:**

We developed immersive technology instructional guides for pediatric health care. The training guides were created for multiple software content and hardware types across several clinical scenarios. Content was available in print and digital versions. The primary outcome was technology use across sites with no-cost software agreements. The secondary outcome was the specific application types used at a single site, stratified by sessions and minutes. Data were analyzed using descriptive statistics.

**Results:**

Data were collected from 19 licensed sites from January through June 2022. Among the 19 sites, 32% (n=6) used 10 or more VR units. Among the 6 sites that had projectors, half used 5 or more units. The mean minutes of use per month of all sites combined was 2199 (IQR 51-1058). Three sites had more than 10,000 minutes of total use during the 6-month review period. Secondary results indicated that active VR (977 total sessions) and passive projector streaming (1261 total sessions) were the most popular application types by session, while active projector (66,849 total minutes) and passive projector streaming (32,711 total minutes) were the most popular types when stratified by minutes of use. The active VR application with the most minutes of use was an application often used in physical therapy.

**Conclusions:**

Context-specific technological instruction coupled to no-cost licenses may increase access to immersive technology in pediatric health care settings*.*

## Introduction

Although immersive technologies are rapidly developing, their adoption in health care greatly varies due to differences in access and acceptability. Recent research has supported novel health care applications of immersive technologies such as virtual reality (VR) [1,2]. Differences in user backgrounds, including technological literacy and financial resources, remain access barriers for health care systems, creating a digital divide [3-5]. Although immersive technologies are not yet widely used, the market is growing quickly due to reduced costs and improved technology [6,7].

Despite the abundance of efficacy research of immersive technologies in health care, standardized strategies for effective implementation across multiple hospital settings are lacking. Immersive technologies may be adopted for a variety of clinical uses, including patient education, surgical planning, and rehabilitation [8-10]. VR also has analgesic properties, with distraction, focus-shifting, and skill-building identified as its mechanisms for reduced pain perception [11-15]. Given the opioid epidemic, the analgesic benefits of VR could be widely implemented as another tool to reduce the morbidity related to opioid misuse [16-20].

Andragogic learning theories provide the foundation for the development of tools to train health care professionals on how to best use immersive technologies [18-21]. Effective adult learning is guided by the principles of cognitive load engagement and active learning [21-24]. Adult learning theories suggest that short videos and multimedia presentations are more effective than traditional didactic lectures [25]. Lengthier video trainings have variable effects on long-term retention, whereas shorter, segmented videos improve recall [21,26,27]. The use of multimedia instruction, including web-based content, further engages adult learners [28,29].

Given the benefits of immersive technologies, we sought to integrate immersive technologies in pediatric health care settings with a standardized set of clinical guides coupled with no-cost software licensing. While developing learning materials, we remained cognizant of the recency of immersive technology, factors that influence immersive technology acceptance, and best practices to improve learning outcomes.

The primary aim of this descriptive feasibility study was to measure the utilization rate of immersive technologies in a variety of pediatric health care settings after the implementation of standardized training with no-cost software licenses. The secondary aim was to explore the types of immersive technology applications used at a single institution.

## Methods

### Context

This study was conducted as part of a research and clinical program at an academic children’s hospital (Lucile Packard Children’s Hospital Stanford [LPCHS]), focusing on the research, development, and validation of immersive technologies for use in pediatric health care [30]. Physicians who lead the program founded a federal, tax-exempt, nonprofit corporation. The mission of the nonprofit is to distribute pediatric immersive technology applications to reduce anxiety, support rehabilitation, and promote pain perception reduction. This nonprofit also helps children with harm reduction, healthy choice education, and mental health support. The nonprofit works with researchers and health care professionals (including but not limited to physicians, nurse practitioners, registered nurses, child life specialists, and physical therapists) to create and distribute free software that is fun, nonviolent, non-nauseating, and practical for many clinical settings. In addition to providing no-cost software licenses, the nonprofit provides training to help these professionals embed immersive technology in clinical practice. Data for this study were collected from January through June 2022.

### Hardware

VR applications distributed by the nonprofit use both portable hardware, including Oculus Go (Meta, Inc), Oculus Quest/Quest 2 (Meta, Inc), and Pico G2 (ByteDance, Ltd). The nonprofit also distributes projector-based applications that use the Nebula Capsule (Anker Innovations Co), a portable smart projector that displays visuals on a surface secured with a mounting clip [31,32].

### Training

**Figure 1:**
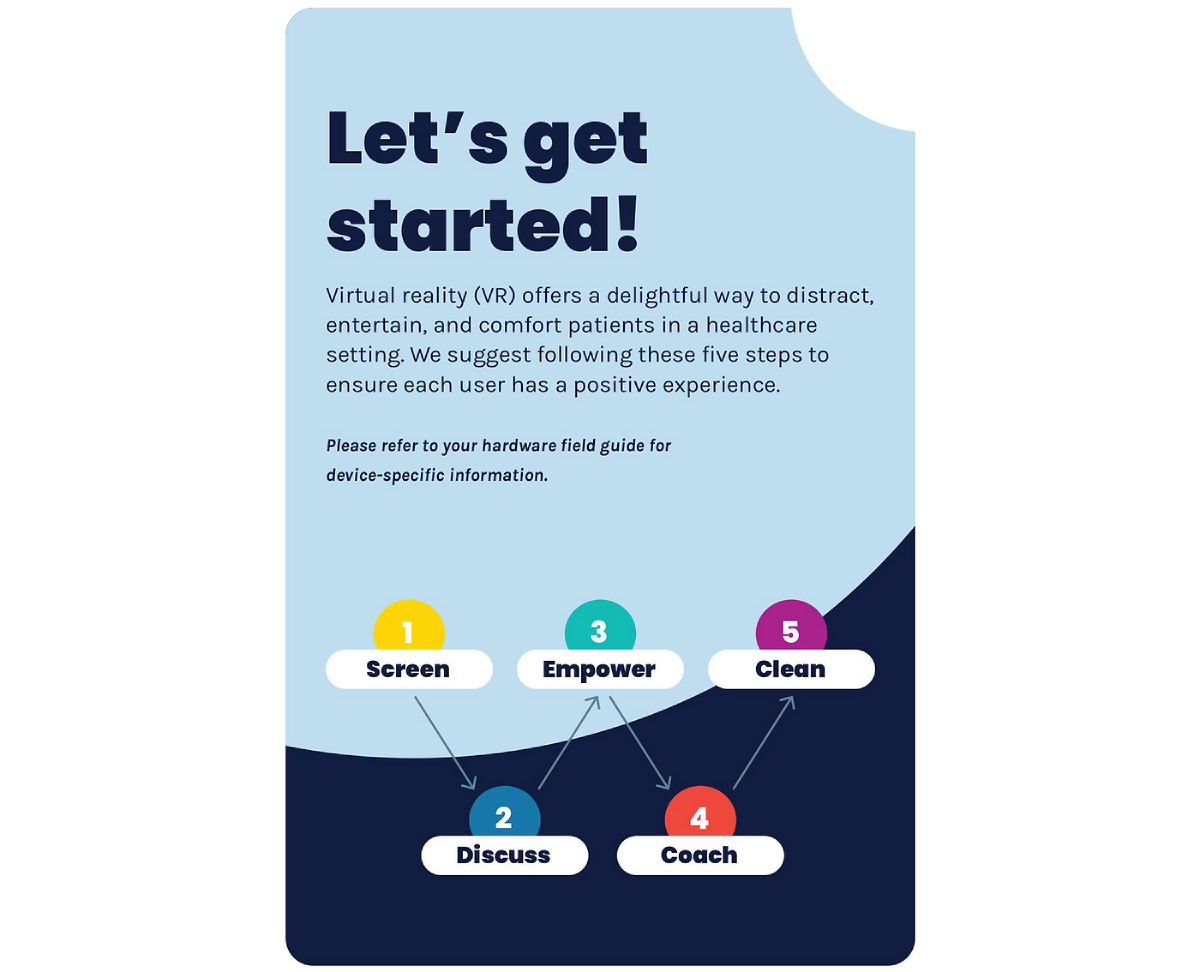
Excerpt from a learning guide.

The training consisted of a series of instructional videos and step-by-step written instructions, available in print and in digital, web-based format. The nonprofit developed a novel framework for introducing and guiding pediatric patients through VR experiences. This framework contained 5 steps: screen, discuss, empower, coach, and clean (Figure 1). These 5 steps were adapted from adult learning theory to fit a health care context, the specific needs of a pediatric population, and the constraints of immersive technology experiences. The nonprofit designed the intervention to be sensitive to several factors: the cognitive load required of practitioners to learn a new skill in a high-risk environment, the plurality of pediatric patient health care needs and constraints, and the requirement to present immersive technologies to patients in a way that elicited their participation.

First, to remain sensitive to the occupational demands on practitioners’ working memories, any intervention would need to respect these demands by the use of a sufficiently scaled information hierarchy. For example, the intervention would need to make only the vital information quickly accessible and only deliver new information as necessary. This was accomplished by creating separate guides according to use case, revealing actionable steps around a broadly applicable framework, and through a nested information hierarchy in the digital intervention materials. We further reduced the cognitive load on practitioners by prescribing as many directives for actions and sentence frames for dialogue as possible without tailoring those prescriptions too narrowly to a specific use case.

Second, the intervention needed to balance ease of accessibility with sufficient nuance to capture diverse patient needs. The content catered to the disparate uses of immersive technologies (ie, physical therapy, distraction from pain or fearful environments, and anesthesia induction), patient characteristics (ie, age, mobility, cognitive ability, level of comfort with VR, quality of eyesight, body position requirements of certain procedures), and the needs of specific experiences (ie, dialogue prompts, experience-specific movements, level of difficulty).

Third, the intervention was designed to elicit patient engagement. Leveraging self-determination theory, the intervention emphasized agency and choice for patients in terms of their VR experience and the amount of information about the external environment they wanted to receive during the VR experience [33,34]. This was also accomplished by incorporating multiple decision points after receiving new information about the VR experience.

The medium consisted of static digital guides that could be printed or digitally referenced, video guides that demonstrated how to conduct a VR experience with patients with different health care goals in varied contexts, and a hierarchically nested digital library that included all resources in addition to descriptions of each immersive technology and their respective use case (Multimedia Appendix 1). Video guides detailed (1) how to use the equipment, (2) how to present the immersive technology intervention to patients, and (3) how to conduct each immersive experience (Multimedia Appendix 2). The guides were available to all licensed nonprofit users.

### Outcomes and Measures

The primary aim of this study was to measure the utilization rate of immersive technologies at institutions that licensed the software through the authors’ nonprofit. The secondary aim was to analyze the use of different types of immersive technology applications at a single institution according to site-specific data. Applications were stratified by VR, projector, active, passive, or training. Use was measured by the number of sessions launched and total number of minutes engaged in an application. These data were not available from the entire cohort due to technological privacy related to the license.

Anonymized data were collected from a mobile device management dashboard (Manage XR). This allowed for the measurement of the type of applications (active vs passive), the length of time for which the application was used, and the number of sessions launched.

### Analysis

Descriptive statistics were used to analyze the use of immersive technologies at the index institution (LCPHS) as well as at the sites to which the technologies were distributed. Results are reported as means and IQRs.

### Ethical Considerations

The Stanford University Institutional Review Board provided a waiver of the requirement of approval owing to the use of historical data.

## Results

### Primary Outcome: Institutional Use

**Figure 2:**
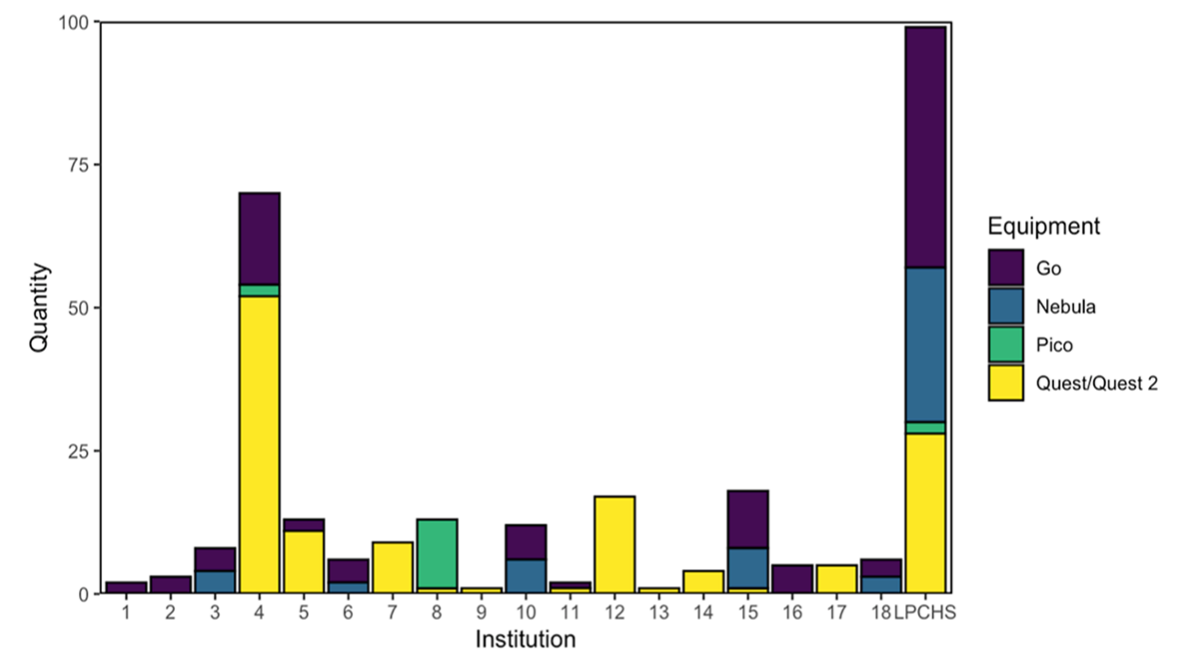
Tye of immersive technology equipment available across sites. LPCHS: Lucile Packard Children’s Hospital Stanford.

The software was licensed to 18 institutions in addition to LPCHS. Institutes were located across all 4 Census Bureau regions of the United States (4 in the Northeast, 4 in the Midwest, 5 in the South, and 3 in the West), in addition to 3 sites in Canada. The majority of users seeking license and training agreements were first-time or novice users*.* Although most (n=17) sites had fewer than 25 pieces of equipment, site 4 and LPCHS possessed over 50 types of equipment. Go and Quest/Quest 2 were the most commonly used equipment types at 34% (100/297) and 44% (131/297) of equipment totals, respectively (Figure 2). Thirteen sites did not have Nebula projectors available; all 6 sites that did have these projectors had at least the same number of VR devices as Nebula projectors (VR/projector ratio>1).

**Figure 3:**
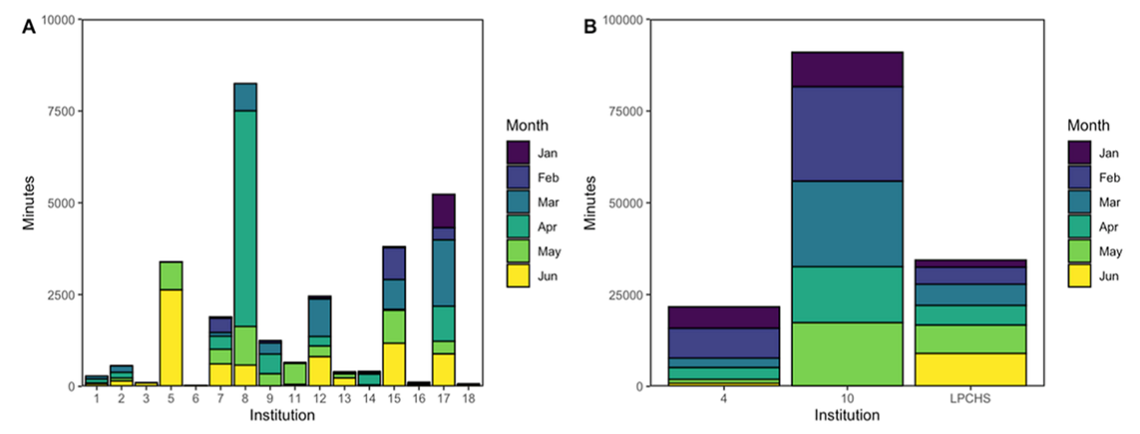
(A) Usage minutes over the review period by month and site for sites with ≤10,000 total minutes of use. (B) Usage minutes over the review period by month and site for sites with >10,000 total minutes of use. LPCHS: Lucile Packard Children’s Hospital Stanford.

Equipment was variably used among institutions (Figure 3A-B). The mean of monthly usage was 2199 (IQR 51-1058) minutes across all sites combined (Figure 3A). Sites 4, 10, and LPCHS were notable for substantial use with an average of 3613 (IQR 1443-5202), 18,200 (IQR 15,249-23,293), and 5734 (IQR 4807-7252) monthly minutes, respectively (Figure 3B). Only 3 sites had more than 10,000 minutes of total usage across the review period.

### Secondary Outcome: Types of Applications Used

**Figure 4:**
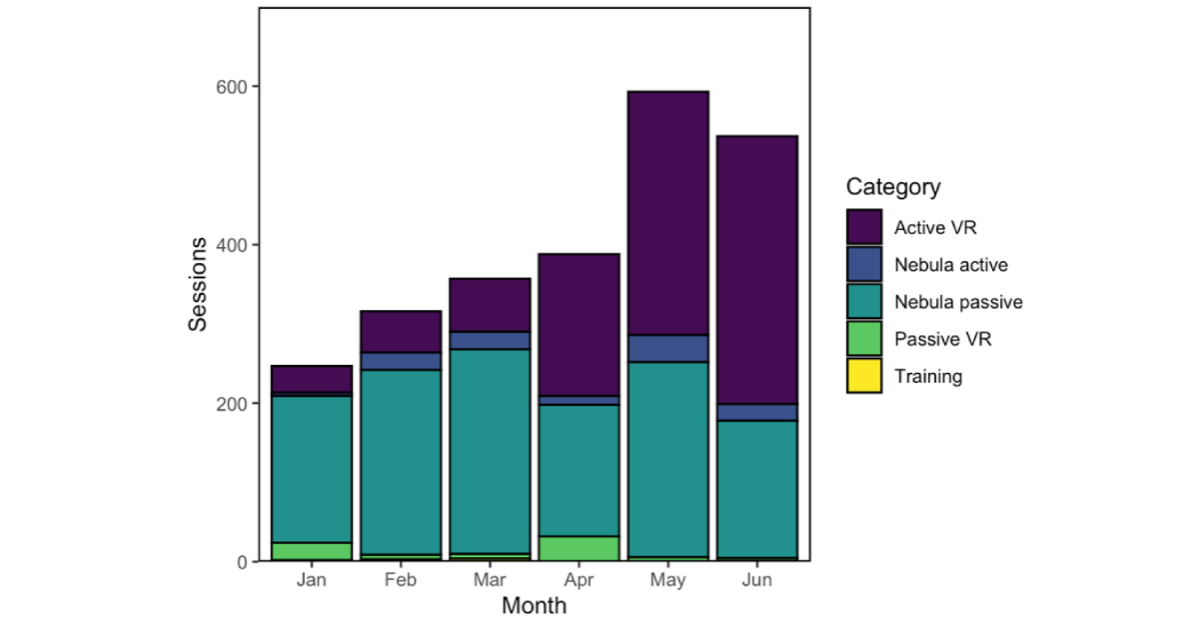
Types of applications at Lucile Packard Children’s Hospital Stanford. VR: virtual reality.

The number of sessions and stratified application type increased over the review period at LPCHS (Figure 4). While the level of passive projector use remained consistent, there was an increase in active VR usage. Patients used active VR and passive projector media applications most commonly when analyzed by session count, with 977 and 1261 total sessions, respectively (Figure 4). Patients used active and passive projector streaming most commonly when usage was analyzed by minutes, with 66,849 and 32,711 minutes of use, respectively.

**Figure 5:**
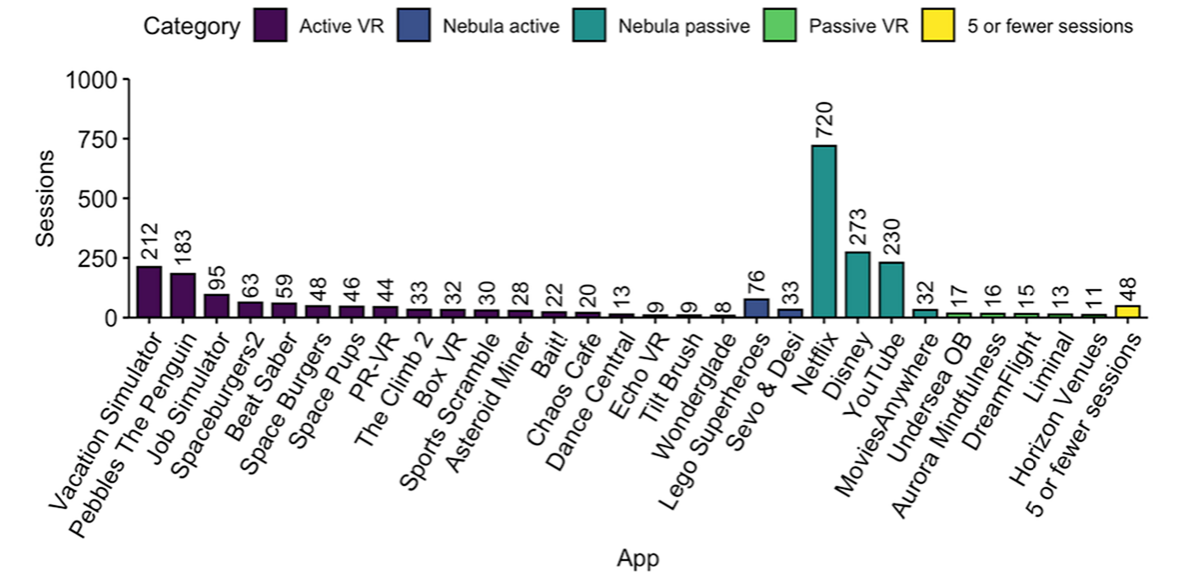
Sessions per application at Lucile Packard Children’s Hospital Stanford. VR: virtual reality.

Analysis of specific applications revealed that most sessions of active VR applications were games, including Vacation Simulator (Owlchemy Labs), which accounted for 22% (212/977) of all active VR sessions. The most-launched passive projector sessions were streaming services such as Netflix, accounting for 57% (720/1261) of all passive projector sessions (Figure 5).

**Figure 6:**
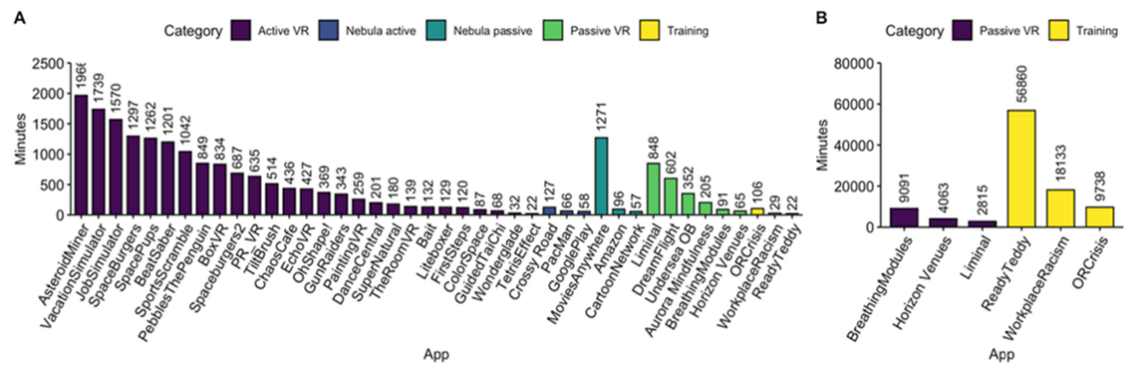
(A) Minutes of use per application with ≤2500 total minutes at Lucile Packard Children’s Hospital Stanford (LPCHS). (B) Minutes of use per application with >2500 total minutes at LPCHS. VR: virtual reality.

The active VR application with the most minutes of use was an application often used to promote rehabilitation called The Climb 2 (Crytek) with 2815 minutes of use, accounting for 15% (2815/19355) of all active VR minutes. The most frequently used active projector application by minutes was an application designed to facilitate anesthesia induction called Sevo & Desi (Stanford Chariot Program), accounting for 85% (56,860/66,849) of active projector minutes. The most frequently used passive projector application was YouTube, accounting for 55% (18,133/32,711) of passive projector minutes (Figure 6A-B).

## Discussion

### Principal Findings

Immersive technologies can be widely disseminated with no-cost licenses and complementary training materials. This study identified variation in the quantity and format of immersive technologies used across each site. The extent of immersive technology use appears to be influenced by the amount of equipment available at each site; most sites had fewer than 25 units of equipment available.

Different hardware is used for different clinical applications in children of different ages. For example, portable VR units may be used for acute distraction at the bedside in older children, whereas projectors are ideal for distraction during patient transport, particularly for younger children. Owing to the different clinical uses among sites, we observed variations in the types of immersive technology units deployed at each site. For example, some institutes only had Go units, such as sites 1, 2, and 16. Many other sites did not have projector units available, limiting streaming options for patients. Most sites were lacking one or more equipment types. Only LPCHS was equipped with all 5 equipment types.

We did not observe any generalizable trends in usage minutes across sites during the 6-month observation period. Rather, monthly usage minutes tended to fluctuate. However, sites with greater monthly minutes tended to experience consistently greater usage across all months than sites with lower usage minutes. At LPCHS, active VR and passive projector media streaming applications were used more often than active projector, passive VR, and training applications, potentially due to the user reception and clinical use setting. Although the data lack information on clinical context, active VR may have been selected by health care professionals when patients needed more immersion and engagement, such as during a stimulating procedure. Technological instruction for hospital staff may have contributed to the increased use of active VR applications at LPCHS, which generally require more training than passive modalities.

Further analyses of the types of software applications used in the hospitals would complement the LPCHS institutional data. Of all application types, we observed the greatest use of active VR and passive projector content. Whereas active VR and active projector applications generally consisted of a mix of content created by the LPCHS research program and commercially available applications, passive projector applications were more likely to be commercially available streaming services. There is a broad selection of immersive technology content available and further research will be needed to identify the optimal content for different clinical scenarios.

### Strengths and Limitations

This study demonstrates the promise for the multi-institutional use of immersive technologies through evidence-based instructional methods. Numerous human factors studies have demonstrated similar outcomes in different domains, including agriculture, industrial organization, household technology use, and online learning [35-38]. This study extends these outcomes by demonstrating the feasibility of the widespread adoption of immersive technology within the pediatric health care setting. Using a teaching model that prioritizes efficient integration and user accessibility may be a key factor to bridge technology research and clinical use. Furthermore, when institutions are provided with technical instructions based on adult learning techniques, adoption may be improved.

This study had several limitations. First, prior to adoption, all users would have reported their familiarity with device use to better quantify the training effectiveness. However, given the primary goal of increasing use and the deleterious effects of surveys on motivation, we opted to not include a preassessment.

Second, privacy regulations and protections on patient information limited our ability to determine the clinical context associated with each instance of immersive technology use. Additionally, outside of LPCHS, the number of sessions and types of applications were not available for analysis due to privacy policies.

Third, while data on patient demographics and specific uses of each application would have increased our understanding of VR utility, the aim of this project was to demonstrate wide-scale use. The hardware and software licenses did not provide access to protected health information to ensure the cyber safety of users. It was outside the scope of this project to have research assistants collect information on patient and use contexts at the wide variety of institutes included. Unlike most VR studies that demonstrate specific health care uses without attention to practical implementation, this study describes a means toward practical implementation*.* Despite the lack of an entire cohort of institutional data, the types of applications used at LPCHS provide important information on the natural clinical use of immersive technologies in a pediatric hospital.

Lastly, while we successfully identified and analyzed widespread adoption across sites, we were unable to obtain a staff-level assessment of user sentiments or demonstrate causality between training materials and utilization rates.

### Future Directions

This study demonstrates that technological digital instruction can facilitate the use of immersive technology in a wide variety of pediatric health care settings. Such methods have the potential to increase user acceptability and to be adapted to the instructional context. Further research will focus on the efficacy of different educational tools during technology instruction with user-specific feedback. Additional efforts will be made to evaluate factors that influence the acceptability of immersive technologies through the customization of training methods at institutions.

## Appendix

Multimedia Appendix 1 Digital app library materials (excerpt from website).

Multimedia Appendix 2 Virtual reality physical therapy field guide (excerpt from website).

## Abbreviations

LPCHS: Lucile Packard Children’s Hospital Stanford
VR: virtual reality

## Data Availability

The data sets generated and/or analyzed during this study are available from the corresponding author on reasonable request.
