# Supplementary figures and images for: Enhancement of Immersive Technology Use in Pediatric Health Care With Accessible, Context-Specific Training: Descriptive Feasibility Study
Source: JMIR XR Spat Comput. 2024 Jul 30;1:e56447. doi: 10.2196/56447 (PMC12671333; doi:10.2196/56447)

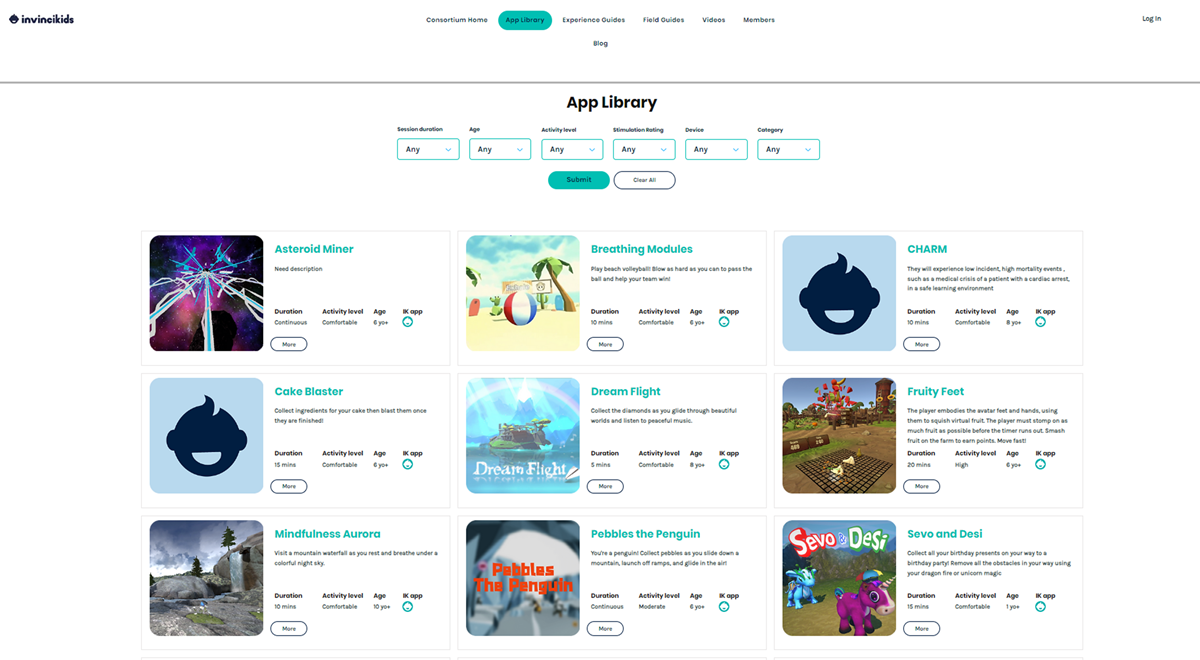

Supplement: Multimedia Appendix 1 [file xr_v1i1e56447_app1.png]
